# Inhibited radiative decay enhances single-photon emitters

**DOI:** 10.1038/s41467-026-75489-5

**Published:** 2026-07-16

**Authors:** Florian Burger, Stephan Rinner, Andreas Gritsch, Kilian Sandholzer, Andreas Reiserer

**Affiliations:** 1 Technical University of Munich, TUM School of Natural Sciences, Physics Department and Munich Center for Quantum Science and Technology (MCQST), Garching, Germany; 2 TUM Center for Quantum Engineering (ZQE), Garching, Germany; 3 Max Planck Institute of Quantum Optics, Quantum Networks Group, Garching, Germany

**Keywords:** Single photons and quantum effects, Quantum information

## Abstract

Quantum networks and modular quantum computers require efficient spin-photon interfaces, often realized using optical resonators that enhance radiative decay on a desired transition. However, this requires small mode volumes and high quality factors, which limits multiplexing capacity and demands precise frequency tuning. Here, we demonstrate an alternative approach that circumvents these bottlenecks for upscaling. Using a W1 silicon photonic-crystal waveguide with a tailored photonic bandgap, we selectively inhibit unwanted decay pathways, thereby redirecting emission to the desired transition. This enables efficient photon collection over a large frequency range, allowing the resolution and individual addressing of tens of erbium dopants. Their lifetimes are preserved, or even increased, compared to bulk material. The extended mode volume of the devices enables the use of lower dopant concentrations, thereby improving emitter coherence. Our approach can be combined with Purcell enhancement and applied to other spin-qubit platforms, opening intriguing perspectives for photonic quantum technologies.

## Abstract (web summary)

Spin–photon interfaces for quantum networks often rely on resonator enhanced emission, limiting scalability and requiring precise tuning. Here, the authors demonstrate an alternative approach that avoids these limitations by suppressing unwanted radiative decay channels of erbium dopants using silicon photonic crystal waveguides.

## Introduction

**Fig. 1:**
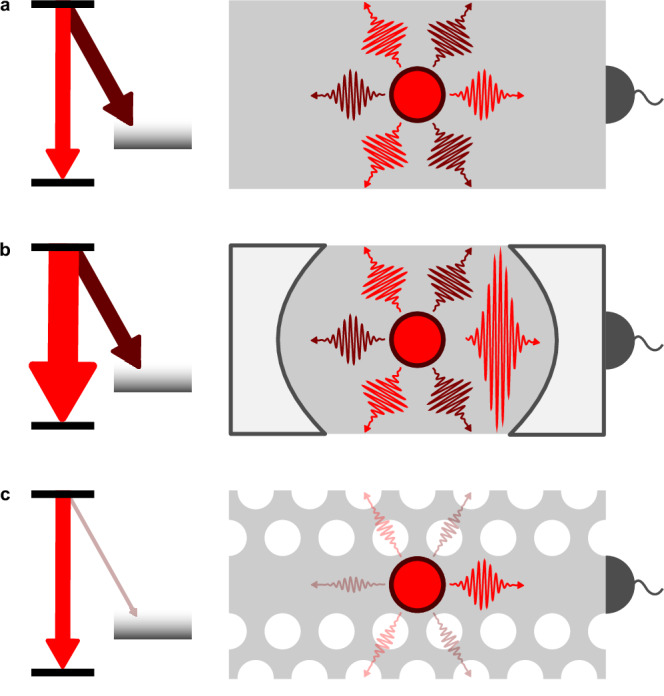
Different approaches to interfacing single-photon emitters. **a** An emitter (filled red circle) in a homogeneous medium (gray) emits photons (colored wave packets) in many directions and on desired (light red) and undesired (dark red) optical transitions (arrows in the level scheme on the left). Thus, only a small fraction of the photons is emitted on the desired transition, collected and detected, e.g., with a fiber-coupled single-photon detector (black symbols on the right). This hampers the performance as a single-photon source. **b** Integrating the emitter into a suited optical resonator (indicated by the two mirrors) can strongly enhance the desired transition (large red wave packet) via the Purcell effect. However, the emission into other spatial modes and on other transitions is largely unaffected. Therefore, such single photon sources require strong Purcell enhancement factors and tight light confinement to be efficient. **c** In contrast, the emission into all but one spatial and spectral mode can be strongly suppressed, e.g., by photonic crystal waveguides (gray). Such devices can have an increased bandwidth and do not require precise frequency tuning and stabilization. They are not restricted to small mode volumes and thus their spectral multiplexing capacity is not limited by emitter interactions.

Controlling the generation of single photons by solid-state emitters^1^ is key to scaling up photonic quantum technologies. In an ideal system, all photons should be emitted into a single mode of space, time and frequency. However, all physical systems explored so far—including trapped atoms, color centers, quantum dots, molecules, and rare-earth dopants—exhibit undesired optical decay channels that deteriorate their performance as single-photon sources. Depending on the platform, these unwanted transitions can involve phonons, other states of multilevel emitters, and spatial modes that cannot be collected efficiently (Fig. 1 a).

In many previous experiments, this limitation has been overcome by embedding the emitters into optical resonators that enhance the wanted transition via the Purcell effect^2^ (Fig. 1 b). However, this approach also has several downsides: First, achieving a sufficient Purcell enhancement factor *P* requires resonators with a small mode volume. Thus, dipolar interactions may hamper the multiplexing of many emitters in a single device. In addition, high *P* is only achieved in resonators with high quality factors, which restricts the bandwidth and requires sophisticated resonator fabrication and tuning techniques. Finally, high values of *P* can lead to very short emitter lifetimes, which is a double-edged sword. On the one hand, the resulting linewidth broadening can be beneficial for overcoming the challenges posed by spectral instabilities of the emitters^2^. In addition, in short-distance connections, short lifetimes may enable a speed-up, in particular when using rare-earth dopants. On the other hand, long-distance scenarios are limited by the two-way signaling time, which, e.g., approaches ms timescales for typical distances of 100 km. In this situation, lifetime reductions are typically not required to increase the success rate but may unnecessarily increase the demands on the experimental control and detection system while restricting spectral multiplexing capacity^3^. Also, for fast emitters, further reducing the lifetime may increase two-photon emissions in resonant excitation^4^, which hampers most applications in distributed quantum information processing.

In this study, we therefore explore an alternative concept. Instead of enhancing a desired transition using a resonator, we use the concept of inhibited spontaneous emission^5,6^ to suppress the unwanted radiative transitions (Fig. 1 c). The underlying approach has been studied previously in different solid-state systems. It has been shown that the lifetime^7^ and the emission spectrum^8^ of emitters in nanocrystals depend on their size and surrounding refractive index. Similarly, the ratio of electric and magnetic dipole emission in rare-earth doped thin films in proximity to a gold mirror has been investigated^9^. Additionally, integrating solid-state emitters into nanostructured environments^6^ has enabled advanced control over the optical local density of states (LDOS)^10^ in waveguides^11^ and photonic crystals^12,13^. This has allowed enhancing the emitter decay into a single spatial mode while reducing the radiative lifetime, achieving near-unity beta factors with quantum dots^14^ and color centers in diamond^15^.

Here, instead of tailoring the LDOS such that the emission is increased, we explore the potential of inhibiting the spontaneous emission on undesired radiative transitions. Specifically, we study erbium emitters in silicon photonic-crystal waveguides (PCWs). By reducing the LDOS over a broad spectral range, we implement an efficient, broadband, and multiplexed single-photon source in the telecommunications C band in which the emitter lifetime is not reduced but preserved or even increased. As the studied effect is largely independent of the waveguide length, our approach enables multiplexing many emitters at low concentration, potentially overcoming concentration-dependent limitations on the spectral stability of erbium dopants^16,17^.

Our experiment is based on erbium dopants in nanophotonic silicon waveguides with a large band gap that reduces the optical density of states over a broad spectral range. Erbium-doped silicon has recently emerged^18^ as a promising hardware platform for quantum networks that combines coherent optical transitions^19,20^ in the minimal-loss band of optical fibers^21^ with millisecond ground-state spin coherence^22^. Furthermore, it is fully compatible with foundry-based semiconductor manufacturing^23^. When integrated into optical resonators, the lifetime of the optical transitions can be strongly reduced^16^, and individual spins can be controlled and read out in a single shot^24^. However, the small volume and narrow bandwidth of the resonators used in these experiments have restricted the spectral multiplexing capacity to a few dopants. This limitation is overcome in our current approach. We can thus spectrally resolve, individually address, and study tens of single emitters in the same device.

## Results

### Photonic crystal waveguide design

**Fig. 2:**
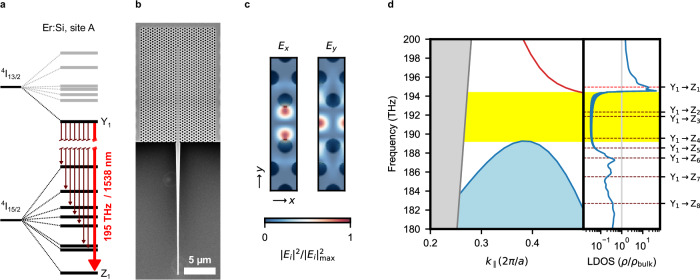
Photonic crystal waveguide (PCW) for tailoring the radiative decay of individual erbium dopants in silicon. **a** Level scheme. The interaction with the host crystal splits the spin-orbit coupled energy levels ^4^I_15/2_ and ^4^I_13/2_ of erbium’s 4f electrons into the crystal field levels *Z*_1_ to *Z*_8_ and *Y*_1_ to *Y*_7_, respectively. At cryogenic temperatures, the *Y*_1_ and *Z*_1_ levels cannot decay via phonon emission and thus exhibit long optical coherence. **b** W1 waveguide. The scanning electron microscope image shows a representative PCW terminated by a tapered fiber coupler at the bottom and a photonic crystal mirror at the top. **c** Normalized absolute value squared of the strongest electric field components, *E*_*x*_ (left panel) and *E*_*y*_ (right), of the guided eigenmode in a unit cell of the W1 waveguide. **d** Simulated photonic band structure (left) and LDOS (right) for a *y* dipole at the maximum field position of *E*_*y*_. Above the line where *ω*/*k*_∥_ equals the speed of light (gray area), the spectrum of states is continuous. Below, a photonic band gap is formed, surrounded by slab modes (light blue) with reduced LDOS. Along the *Γ* − *K* direction (*k*_∥_), two modes are guided in the W1 waveguide with lattice constant *a*. Below the lower, even mode (red), an approximately 5 THz wide gap (yellow) remains. Thus, the LDOS is significantly reduced for the transitions *Y*_1_ → *Z*_2_. . . *Z*_8_, (right axis) of embedded erbium dopants, suppressing their spontaneous emission. In contrast, on the Y_1_ → Z_1_ transition, the slow light effect leads to an increased LDOS and an enhanced decay into the guided mode.

In this work, erbium is integrated into silicon at lattice site “A”^25^, for which the transitions between the lowest crystal field (CF) levels *Y*_1_ and *Z*_1_ of the *I*_15/2_ and *I*_13/2_ manifolds occur within the telecommunications C band at ~1538 nm (Fig. 2 a). In bulk silicon, this transition has a small branching fraction of 23(5)%^19^, meaning that the *Y*_1_ state decays predominantly to the higher CF levels with wavelengths between 1550 and 1650 nm. This work aims to eliminate these unwanted decay channels, so that the excited state decays predominantly via the *Y*_1_ → *Z*_1_ transition into a single waveguide mode efficiently coupled to an optical fiber.

For that purpose, we use PCWs that feature a large photonic band gap (PBG) and a strongly dispersive guided mode. The starting point is a photonic-crystal slab, a quasi-2D photonic crystal, consisting of a triangular lattice of cylindrical air holes in an undercut, high refractive-index membrane of sub-wavelength thickness. An SEM image of such a device is shown in Fig. 2 b. Leaving out a single row of air holes in the *Γ* − *K* direction creates a W1 waveguide^26^ that features two guided modes, one with even (shown in Fig. 2 c) and one with odd symmetry. The surrounding photonic crystal slab has a 50 THz wide PBG for TE-like modes. Fig. 2 b shows the photonic band structure for a PCW of infinite length, which we compute using a frequency-domain eigensolver^27^. The devices studied experimentally exhibit the same cross-section but different finite lengths (see methods). The PBG in between the slab modes (light blue) extends approximately from 190 THz to 240 THz. The even mode (red) at lower frequencies features a flat dispersion relation at the edge of the Brillouin zone, indicating “slow light”, i.e., propagation with a strongly reduced group velocity^28,29^. In the region between approximately 190 THz and 195 THz, a TE band gap remains (yellow), suppressing all emission at these frequencies.

Alongside the band structure, we show the normalized LDOS in a symmetric PCW that is open at both ends. It was simulated using a finite-difference time-domain method^30^ for an emitter with an in-plane electric dipole perpendicular to the waveguide direction (*y* dipole) at the *E*_*y*_ field maximum of the guided mode (cf. Fig. 2 c). The LDOS shows a strong spectral dependence^31^: In the frequency range of the even guided mode (red in Fig. 2 d), it is equal to or greater than one (gray line), and it increases towards the edge of the band gap (*ρ*/*ρ*_bulk_ > 10) as the group velocity tends to zero^10,32^. Thus, the radiative decay of transitions in this frequency range is enhanced via the Purcell effect. In practice, the maximum achievable Purcell enhancement is limited by finite size effects^33^ and ultimately by fabrication imperfections^34^. In the band gap, between approximately 190 THz and 195 THz, emission is strongly suppressed (*ρ*/*ρ*_bulk_ < 0.1). At lower frequencies, where only unguided slab modes are available, the suppression is weaker but still considerable (0.1 < *ρ*/*ρ*_bulk_ < 1).

In addition to the spectral dependence, the LDOS exhibits a spatial variation: The Purcell enhancement is most substantial at the electric-field maximum and vanishes at positions with a small field. Fig. 2 d only shows the LDOS at the maximum of the electric field component *E*_*y*_ for a dipole oriented parallel to the field; the spectra for other dipole orientations and positions in the investigated structures are presented in Sec. I of the supplementary information. There, one can see that independent of the specific position and orientation in the waveguide, the emission of erbium dopants on all unwanted transitions from the optically excited state *Y*_1_ to the higher-lying crystal-field levels of the ground-state manifold (*Z*_2_ to *Z*_8_) will be strongly suppressed. This inhibition of spontaneous emission leads to an increase in the lifetime of the *Y*_1_ state.

The effect is counteracted for dopants at the field maximum whose dipole matches the electric field orientation, such that the emission on the *Y*_1_ → *Z*_1_ transition is enhanced. By choosing suitable parameters for the geometry of the photonic crystal waveguide, the enhancement of the *Y*_1_ → *Z*_1_ transition can be precisely adjusted to compensate for the inhibition of the other transitions. In this way, the emission can be channeled almost entirely into the desired mode without changing the lifetime of the emitters.

### Spectral properties of erbium dopants in a silicon photonic-crystal waveguide

**Fig. 3:**
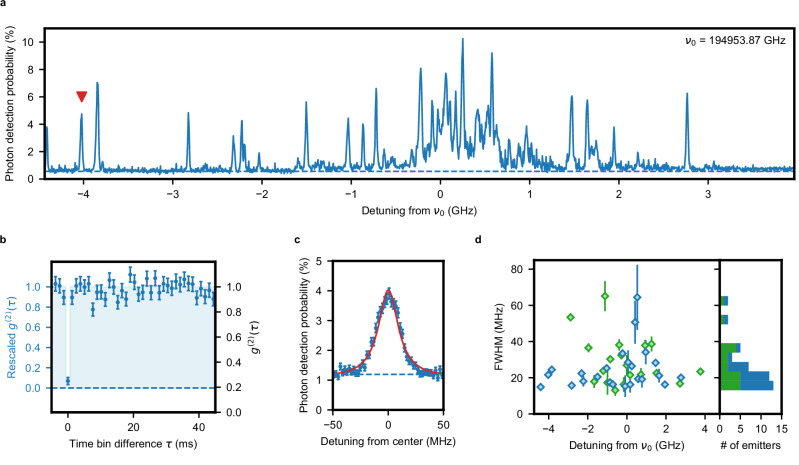
Spectral properties of erbium dopants in a silicon photonic-crystal waveguide. **a** After pulsed resonant excitation around the *Y*_1_ → *Z*_1_ transition frequency *ν*_0_ of site “A”, the fluorescence spectrum of an inhomogeneously broadened ensemble of erbium dopants in PCW A exhibits distinct peaks. The dashed line indicates the dark count level. **b** A measurement of the autocorrelation function *g*^(2)^(*τ*) on the same emission line results in a zero-delay value of 0.07 ± 0.01 when subtracting^60^ the contribution of the independently measured detector dark counts (left axis), or 0.25 ± 0.03 without correction (right axis). This proves that the peak originates from a single dopant. **c** A Lorentzian (red) fitted to a high-resolution measurement of a single emission line (marked by the red triangle in a) gives a linewidth of 21.5(1) MHz. Error bars: 1 S.D. **d** The spectral diffusion linewidth measured on PCWs A and B (blue and green, respectively) slightly differ between the emitters. It exceeds 13 MHz for all dopants. The average linewidth is 27 MHz with a standard deviation of 12 MHz. The error bars indicate the standard error of the Lorentzian fits.

To demonstrate the described tailoring of the radiative decay, we perform pulsed resonant fluorescence spectroscopy^16^ on three different PCWs on the same chip, labeled A, B, and C, that have identical design parameters except for their different lengths (see methods). The devices are mounted in a closed-cycle cryostat at 1.2 K. The dopants are repeatedly excited by laser pulses of 2 μs duration while the excitation laser frequency is swept. After each pulse, the fluorescence is detected by a single-photon counter. Averaging over 8000 repetitions at each frequency setting, we obtain the fluorescence spectrum in Fig. 3 a. The implantation dose of 1 × 10^11 ^cm^−2^ was chosen such that we expect 40 emitters in each waveguide when assuming an integration yield of 1% in site A^19^. Approximately thirty spectrally separated peaks are observed around the corresponding optical transition frequency.

To demonstrate that the peaks originate from single dopants, we measure the autocorrelation function of several of the isolated lines and consistently obtain values of *g* ^(2)^(0) < 0.5, see e.g. Fig. 3 b. In a high-resolution measurement, Fig. 3 c, the emitters exhibit Lorentzian line shapes (red fit curve), approximately four orders of magnitude broader than the lifetime limit. Fig. 3 d shows the extracted linewidth of all peaks; the individual fits are displayed in the supplementary information. The width slightly differs between the emitters, with a minimum value of approximately 13 MHz that is comparable to previous results in nanophotonic resonators^16,24^. As the homogeneous linewidth in Er:Si is much narrower, down to 10 kHz at the temperature of the present experiment^19^, the broadening is attributed to spectral diffusion that originates from charge or spin noise in the devices^35^.

### Optical lifetime

After studying the spectral properties of the emitters, we now turn to the lifetime of the optically excited state. The waveguides in this study are designed such that the increase in lifetime due to the suppression of the unwanted transitions *Y*_1_ → *Z*_2_. . . *Z*_8_ is approximately balanced by the enhancement of the *Y*_1_ → *Z*_1_ decay for optimally positioned and oriented dopants, leaving the lifetime close to unchanged from its bulk value of 142(1) μs ^19^. This enables us to explore the inhibition of spectral decay channels without compromising the signal-to-noise level, which would be limited by detector dark counts in case the emitter lifetime gets too long. While the suppression is approximately independent of the dopant position (see supplementary information), the enhancement of *Y*_1_ → *Z*_1_ varies significantly with the spatial intensity profile of the guided mode (cf. Fig. 2 b). As the emitters are implanted homogeneously, their random orientations and locations in the waveguide will lead to a statistical spread in the lifetime.

**Fig. 4:**
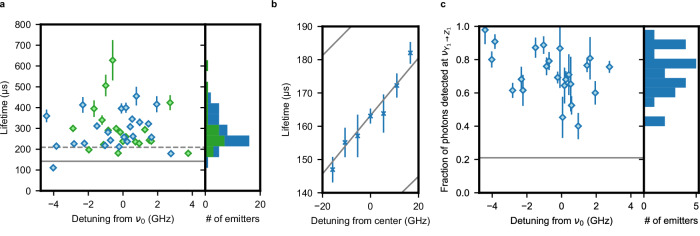
Optical lifetimes and *Y*_1_ → *Z*_1_ emission of single dopants. **a** Exponential fits are used to extract the lifetimes of all individual emitters in the inhomogeneous ensembles of the PCWs A and B (blue and green, respectively), shown as a function of the detuning from the center of the inhomogeneous line. The solid gray line marks the lifetime in bulk silicon of 142(1) *μ*s while the dashed line denotes the value in silicon strip waveguides of 209(1) μs ^19^. The fluctuation of the lifetime, further analyzed in the histogram on the right, stems from the random position and orientation of the emitters relative to the guided mode. The average lifetime is 295 *μ*s with a standard deviation of 97 μs. **b** A magnetic field is applied to tune the emission frequency of a single emitter in PCW C via the Zeeman effect. The spectral dependence of the local density of states (LDOS) can then be determined by extracting the lifetime. The gray curves show the expectation based on the LDOS simulation and the measured dispersion in three different PCWs with parameters similar to those of the device hosting the emitters (see supplementary information). While the slope can be accurately predicted, slight variations across the nanofabricated devices lead to a deviation of the curve offset. **c** As an effect of the selective suppression of unwanted optical transitions, the fraction of the photons that are emitted on the *Y*_1_ → *Z*_1_ transition is increased. It can thus exceed the value measured in a silicon strip waveguide of 23(5)% (gray line). Inserting a narrowband spectral filter allows determining the fraction of light that is detected at the *Y*_1_ → *Z*_1_ frequency (blue data points). It approaches unity for some emitters but exhibits large fluctuations (histogram on the right) because of the random position and orientation of the emitters in the guided mode of the PCW. The average is 72% with a standard deviation of 14%. The measurement was performed on PCW A. All error bars denote 1 S.D.

To study this effect, we perform a time-resolved analysis of the fluorescence data recorded within 600 μs after each excitation pulse. Figure 4 a shows the lifetimes of the *Y*_1_ level of the emitters in two different waveguides on the same chip, extracted from exponential fits to the fluorescence decay. As expected, the lifetimes in the ensemble vary strongly due to the random emitter integration in the PCW. They range from around 100 μs up to 600 μs. The associated histogram shows an asymmetric distribution in which most dopants have a lifetime between 200 μs and 300 μs. A numerical modeling of the distribution would require precise knowledge of the dipolar transition operators in Er:Si, which is left for future work. A correlation between lifetimes and linewidths is neither expected nor observed.

Furthermore, within the inhomogeneous linewidth, no clear dependence on the detuning of the emitters is observed, proving the broadband nature of our approach. Our observations agree with the expectation from the LDOS simulation, which only shows a small frequency dependence within the spectral range of the inhomogeneous broadening that is obscured by the fluctuations caused by the random emitter orientation and position. However, at larger detunings of tens of GHz, one expects a significant effect of the slope of the dispersion relation on the lifetime.

To investigate this, we apply a magnetic field of up to 3 T that splits the *Y*_1_ → *Z*_1_ transition via the Zeeman effect^25^. In this way, the radiative lifetime of a single dopant can be studied as a function of its detuning from the band edge. The result is shown in Fig. 4 b. The observed change of the lifetime with the emission frequency is consistent with a model based on the simulated DOS and the measurement of the dispersion of three different waveguides (gray curves, see supplementary information for details). In the depicted spectral range, the curve can be approximated by a straight line; this changes on larger scales and when approaching the van-Hove singularity at the edge of the bandgap^10^. Thus, adjusting the detuning from the band edge allows tuning this emitter’s lifetime to precisely match the bulk value or that of another dopant. This paves the way toward two-photon interference with high contrast and thus for spin-photon^36^ and remote spin-spin entanglement experiments^37^.

### Relative increase of the *Y*_1_ → *Z*_1_ emission

The extended excited state lifetimes described in the previous section already indicate that radiative transitions are efficiently suppressed as a result of the tailored LDOS of the PCW. Now, we demonstrate that this suppression is spectrally selective and that the emission is efficiently channeled into the *Y*_1_ → *Z*_1_ transition.

To investigate the spectral dependence of the emission, we insert a tunable narrow-band color filter (FWHM = 0.1 nm) into the detection path and repeat the measurement. We then fit the observed peaks with Lorentzian lines and compare their amplitudes *I*_filter_ to those of the corresponding peaks in the unfiltered measurement *I*_0_ while correcting for the independently calibrated filter transmission *χ*. Fig. 4 c shows the relative fraction of photons that are detected on the *Y*_1_ → *Z*_1_ transition, $I_{\rm{filter}}/\left({I}_{0}\cdot \chi \right)$. As the coupling of the emitted light to the waveguide depends on the position and orientation of the emitters relative to the field maxima of the guided mode, which is random in our experiment, large fluctuations are observed. In the range of the inhomogeneous distribution of emitters in Er:Si, the LDOS is almost constant (see supplement); therefore, the branching fraction shows no significant spectral dependence. For all dopants in the ensemble in PCW A, more than 40% of the detected photons exhibit the desired transition frequency. For some emitters, even values close to unity are reached. For comparison, the horizontal line indicates the branching fraction of erbium in silicon rib waveguides without a tailored LDOS, *P*_*Z*1,bulk_ = 23(5)%, measured using the same technique^19^.

Thus, we find that in the fiber-coupled output, the fraction of light at the desired transition frequency is increased. In principle, this may have two causes: First, the PCW or the fiber-optical setup may act as a spectral filter that transmits light at the *Y*_1_ → *Z*_1_ transition frequency but absorbs or scatters photons at the frequencies of *Y*_1_ → *Z*_2_. . . *Z*_8_, thus preventing them from reaching the detectors. Second, the increase may be due to a change in the branching fraction because of a spectrally selective inhibition of the emission. To exclude that our observations are merely caused by the former effect, spectral filtering, we calculate the expected probability of detecting a photon at the *Y*_1_ → *Z*_1_ transition frequency when assuming that the PCW has no effect on the branching ratio, such that the excited state still decays via the *Y*_1_ → *Z*_1_ transition with the bulk probability of *P*_*Z*1,bulk_ = 23(5)%. The full calculation (see Sec. VII of the supplementary information) assumes that the PCW is lossless, that there is no non-radiative decay, that the emission is coupled into the waveguide with 100% efficiency, and that the emitter is excited with 50% efficiency, which is the maximum value possible upon incoherent excitation, as observed at the used laser power and dopant concentration^17^ (see supplementary information). Even in this best-case scenario, one would expect that a photon is detected after the filter in less than 1.84(43)% of the repetitions of the experiment if the branching were unchanged. This is clearly below the experimental value of 3.41(23)%. Thus, the increased fraction of light detected on the *Y*_1_ → *Z*_1_ transition cannot be explained by mere spectral filtering. Instead, the measurement proves that the inhibition of the radiative decay channels is spectrally selective, and that the branching fraction of the *Y*_1_ → *Z*_1_ transition is increased compared to its bulk value. As this transition is not broadened by subsequent phonon-induced relaxation to lower ground-state CF levels, the single-photon properties of the emitters are improved, which paves the way for quantum photonics applications.

To this end, the efficiency of the photon source will be paramount. In our current demonstration, by correcting for the independently characterized losses of the detection setup (see supplement), we can infer an efficiency of 20(2)% in the on-chip waveguide, and 14(1)% after coupling to a single-mode fiber, comparable to the state-of-the-art of single-photon sources at telecommunications wavelength (cf. Table 2 in reference^21^). The achieved numbers can be further improved by increasing the excitation probability (currently  < 50%) using optical *π* pulses or pulsed excitation to a higher CF level, and by increasing the LDOS of the *Y*_1_ → *Z*_1_ transition by operating closer to the band gap and/or with structures that enable even larger group indices^29,33^.

## Discussion

By suppressing the unwanted radiative decay channels of single-photon emitters, we demonstrate a novel approach to implementing efficient single-photon sources. In contrast to earlier works that used nanophotonic resonators^2^, the larger spatial extent and the spectrally broad emission channeling of photons into the guided mode of PCWs allow for controlled coupling of many emitters while avoiding interactions. Thus, our approach increases the capability for spectral multiplexing, as the number of addressable emitters is only limited by spectral diffusion and by the inhomogeneous width of the emitter ensemble rather than by the spectral width or mode volume of the photonic device. The latter can be changed freely by adjusting the waveguide length without an immediate effect on the LDOS.

Our current work focuses on suppressing unwanted radiative transitions while providing only moderate Purcell enhancement of the desired one via the slow-light effect. However, in some situations, it may still be desirable to shorten the emitters’ lifetimes, e.g., in order to overcome their spectral instability^17^. In this case, the Purcell enhancement may be increased in devices with a higher group index^29^, which may use optimized couplers to the slow light region^33^. This can enable Purcell enhancements comparable to those achieved in nanophotonic cavities^2^ at similar bandwidths while preserving much larger mode volumes and thus multiplexing capacity. In addition, our work may also stimulate novel designs of nanophotonic resonators that combine LDOS enhancements on resonance with broadband inhibition of undesired radiative transitions. However, our approach can also be used to engineer efficient spin-photon interfaces with increased optical lifetime. This can be advantageous in ensemble-based quantum memories that store impinging photons in an optically excited state of the emitters^38^, and in precision spectroscopy and all-optical sensing experiments whose resolution can be limited by the emitter lifetime^39^.

For erbium in silicon, our approach can be combined with recent advances in spin control^24^ to realize a spin-photon interface, where the spin can be read out optically after aligning the radiative dipoles with a vector magnet to increase the cyclicity^40^. Using glide-plane PCWs, chiral spin-photon interfaces^41^ can be implemented to enable, e.g., spin-dependent emission into different directions. The multiplexing capability of the devices may be further increased by suppressing spectral diffusion, which is attributed to spin and charge noise in our samples. Thus, a considerable improvement is expected when applying strong electric fields to ionize charge traps, as demonstrated earlier with quantum dots^42^ and color centers^43^. Still, even without these advances, frequency-multiplexed optical entanglement of remote dopants can be achieved in our devices via tailored rephasing protocols^37^. To this end, it would be beneficial to reduce the fluctuation of the lifetimes observed in our experiments, which can be achieved by spatially selective implantation^44^ and by engineering the band structure to obtain flat bands with constant group index over an extended spectral range^29^. This would open the door to exploring the effects of collective couplings in solid-state light-matter interfaces^32,45^.

While our experiment used erbium dopants in silicon, it is not limited to this combination of emitter and host. Instead, our approach can be directly transferred to many other solid-state emitters, provided their lifetime is not dominated by nonradiative decay channels^35^. This includes rare-earth emitters in various host crystals^3,37,46^, color centers in silicon^47–50^, in silicon carbide^51–53^ and in diamond^54^. In particular, as the bandgap of photonic crystals can span tens of THz ^55^ and thus surpass the frequency of localized and propagating phonon modes, our approach can be used to fully suppress phonon sideband radiation that has limited the rates in many pioneering quantum networking experiments with solid-state emitters^56^. Thus, our demonstration of inhibited spontaneous emission paves the way for the implementation of various photon sources that emit efficiently into a single mode and for robust quantum network nodes that do not require precise resonator tuning and stabilization.

## Methods

### Device design

The W1 waveguides used in this work are formed by leaving out a row of holes along the *Γ* − *K* direction of a triangular lattice of circular air holes in a thin slab of silicon. The slab thickness is 220 nm, the lattice constant used is *a* = 420 nm and the hole radius measured by scanning electron microscopy after fabrication is *r* = 0.28*a*. The photonic crystals studied here all have a finite width of 33 rows, and the waveguides two different lengths of 42 (PCWs A and B) and 19 (PCW C) periods. This short length avoids the complication of Anderson localization observed in longer waveguides with higher group indices^57^. On one end, the PCWs are terminated by reinserting 7 holes into the waveguide, which creates an efficient mirror that ensures all light is emitted into one direction. On the other end, all PCWs feature a short fast-light section with a lower group index to improve the coupling efficiency. This step coupler consists of 4 periods with a stretch factor of 1.07^58^. At the wavelength of interest, this leads to a high transmission of light from the slow-light section to an exponentially tapered 20 μm long fiber coupler^59^. To measure the group index, we fabricate the PCWs in pairs where for one of the waveguides, the interface between the fast- and slow-light sections is made partially reflective by inserting an additional hole. In this way, a Fabry-Perot-like resonator is formed between the end mirror and the reinserted hole. By measuring its free spectral range, the effective group index of the waveguide can be determined^14^. A corresponding measurement of the reflection spectrum is shown in the supplementary information.

### Device fabrication

The devices are fabricated similarly to those used in reference^24^. The starting point is a chip diced from a commercial Czochralski-grown silicon-on-insulator wafer (SOITEC) with a 220 nm thick device layer. Erbium is implanted by Innovion at a dose of 1 × 10^11 ^cm^−2^ and an energy of 250 keV, resulting in a Gaussian depth profile, approximately centered in the device layer with a straggle of  ≈ 20 nm and a peak erbium concentration of 1 × 10^16 ^cm^−3^. The chips were first annealed at a temperature of 500^∘^C with a 1 min ramp duration starting from room temperature and a 30 s hold time. This protocol has previously been found to be the optimal procedure for healing implantation damage without affecting the dopants^23^. The PCWs are then patterned using electron-beam lithography (Nanobeam Ltd., nb5) on a positive-tone resist (ZEP 520A) and subsequently transferred to the silicon device layer by reactive ion etching (Oxford Instruments PlasmaPro 100 Cobra) with fluorine chemistry at cryogenic temperatures. In a final step, the sacrificial layer of silicon oxide is partially removed with hydrofluoric acid, leaving the PCW and the taper for fiber-coupling suspended in air.

### Experimental setup

The sample is mounted in a closed-cycle helium cryostat (ICEoxford 1K DryICE) on a three-axis nanopositioning system (Attocube ANPx312, ANPz101). Resonant fluorescence spectroscopy measurements are performed using an optical pulse setup similar to the one used in reference^24^. The pulses are generated from a continuous-wave laser system (Toptica CTL) using two acousto-optic modulators (Gooch&Housego Fiber-Q). In addition, a single-sideband IQ-modulator (iXblue MXIQER-LN-30) with a bias controller (iXblue MBC-IQ-LAB-A1) is used to enable frequency sweeps over the range of several GHz.

Experimental sequences are implemented using an arbitrary waveform generator (Zurich Instruments SHFSG). For optimized dopant excitation, we use linearly chirped pulses with a length of 2 μs and a chirp-width of 10 MHz. On-chip coupling is achieved using an adiabatic coupler that consists of a 20 μm tapered waveguide contacted to a tapered optical fiber (chemically stripped, 3^∘^ taper angle). Fluorescence emitted by the dopants is separated from the excitation pulse using a beamsplitter with a splitting ratio of 95:5 (Evanescent Optics Inc.). It is detected by a superconducting nanowire single-photon detector (ID Quantique) with 75(5)% detection efficiency. A fast optical switch (Agiltron Ultra-fast Dual Stage SM NS 1 × 1 Switch) is used to prevent blinding the detectors by the excitation pulse. For the filter measurement, we use an electrically tunable optical filter (WL Photonics) with a 0.11 nm FWHM Gaussian-shaped bandwidth.

## Supplementary information

Supplementary Information

Transparent Peer Review file

## Data Availability

The datasets generated and analyzed during the current study are available in the mediaTUM repository (10.14459/2026mp1856417).
