# Supplementary material for: Inhibited radiative decay enhances single-photon emitters
Source: Nat Commun. 2026 Jul 16;17:6250. doi: 10.1038/s41467-026-75489-5 (PMC13376192; doi:10.1038/s41467-026-75489-5)
Supplement: Supplementary file 1 — Supplementary Information [file 41467_2026_75489_MOESM1_ESM.pdf]

Supplementary information: Inhibited radiative decay enhances single-photon emitters

Florian Burger, Stephan Rinner, Andreas Gritsch, Kilian Sandholzer, and Andreas Reiserer*
Technical University of Munich, TUM School of Natural Sciences,
Physics Department and Munich Center for Quantum Science and Technology (MCQST),
James-Franck-Straße 1, 85748 Garching, Germany
TUM Center for Quantum Engineering (ZQE),
Am Coulombwall 3A, 85748 Garching, Germany and
Max Planck Institute of Quantum Optics, Quantum Networks Group,
Hans-Kopfermann-Straße 1, 85748 Garching, Germany
(Dated: June 17, 2026)

I. LOCAL DENSITY OF STATES SIMULATIONS

The local density of states (LDOS) in a photonic-crystal waveguide (PCW) is spatially and spectrally anisotropic. To predict the branching ratio and lifetime of embedded erbium dopants, we simulate the frequency dependence of the LDOS for different dipole orientations and at several positions using a finite-difference time-domain (FDTD) method, specifically the `dft_ldos` function of MEEP [1]. It excites a dipole source at a specified point inside the simulation cell and computes the radiated power by accumulating the Fourier transforms of the electric field. In the band gap of the PCW, where emission is strongly suppressed and only little power is radiated, this method is prone to artifacts in the form of high-frequency oscillations that could only be avoided using prohibitively long simulation times. Instead, we apply a second-order Butterworth lowpass filter with a cutoff frequency of 0.2 times the Nyquist frequency to the simulated spectra to remove the artifacts.

For normalization, the filtered spectra are divided by the simulated LDOS spectrum of a dipole in bulk silicon. The LDOS spectrum in the main text (Fig. 2 d) is simulated for a y dipole at the field maximum of a PCW (cf. Fig. 2 c of the main text) with a total length of 40 periods that includes 4-period step couplers at each end, which connect the PCW to strip waveguides terminated by perfectly matched layers to fully absorb the incident light. This way, the structure exhibits an LDOS spectrum that closely resembles that of an infinitely long waveguide [2].

In the experiment, PCWs with a 31-period slow-light section are used. They are connected to a strip waveguide using a 4-period step coupler on one end. On the other end, the waveguide is terminated by a mirror to be able to collect all fluorescence in reflection. This leads to slightly modified spectra, which are shown in Fig. S1 b for x, y and z dipoles at the maxima of E_y and E_x, respectively. The simulated geometries with the position of the dipole source marked as a red dot are depicted in Fig. S1 a.

Just like in the quasi-infinite PCW, the emission of a y dipole located at the maximum of the E_y field component is enhanced for wavelengths below ≈ 1540 nm, strongly inhibited between ≈ 1590 nm and ≈ 1540 nm and less strongly inhibited above ≈ 1590 nm. The mirror at the end of the waveguide gives rise to a standing wave interference pattern, which leads to an oscillation of the LDOS spectrum that depends on the emitter position, see Fig. S1. The spectral position of the interference minima changes with the exact emitter position and therefore averages out for an ensemble of emitters evenly distributed across the PCW. In our measurements, we did not observe an effect of the finite

* andreas.reiserer@tum.de

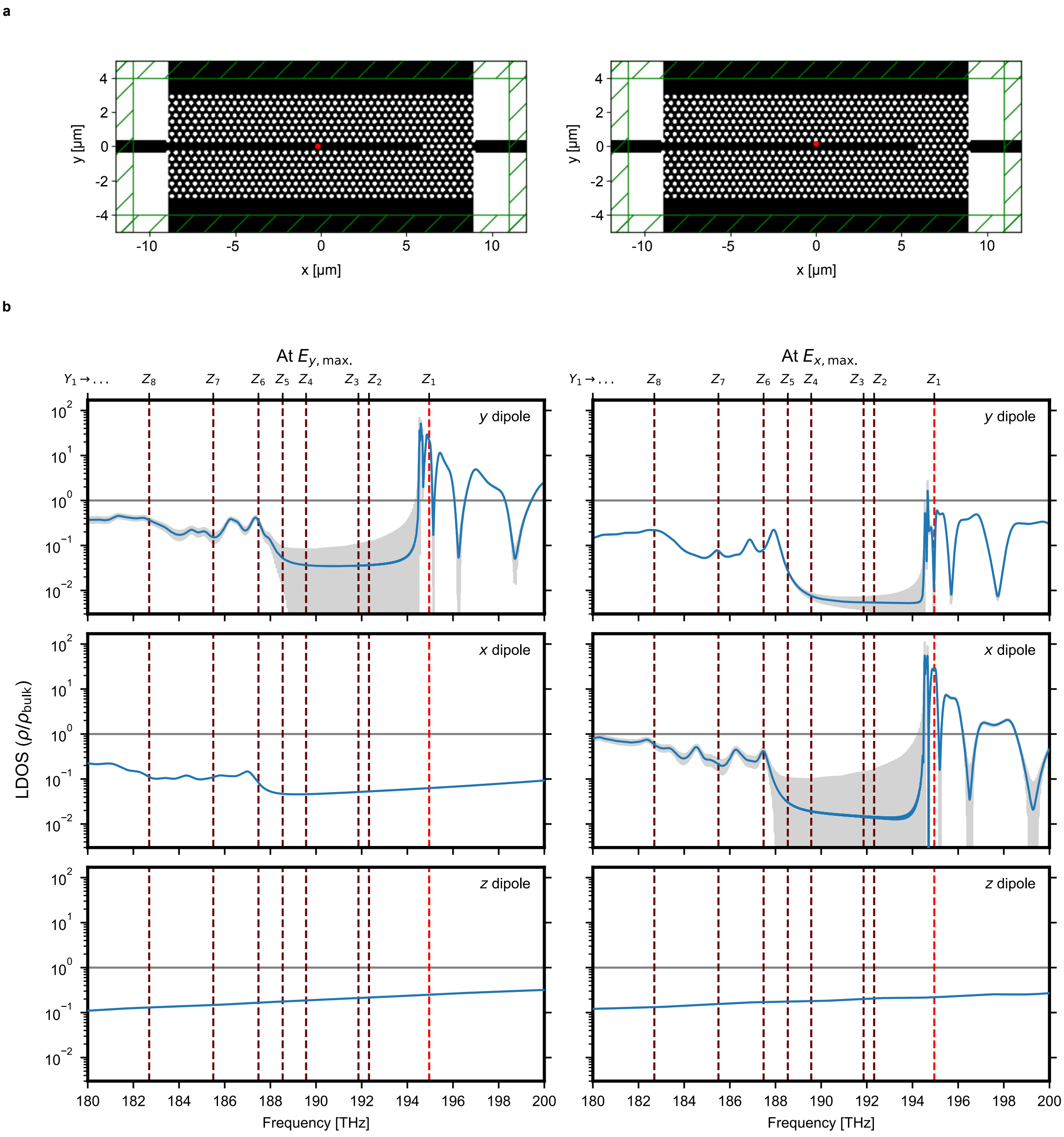

Fig. S1. Local density of states (LDOS) simulation. **a**, PCW geometries used for simulating the LDOS spectra for different dipole orientations at the E_y (left) and E_x (right) maximum. The images show a top view of the photonic crystal slab. The black parts are silicon with a refractive index of 3.45 (at $T < 10\,\mathrm{K}$), the white parts are air with a refractive index of 1. The dashed green area indicates perfectly matched layers (PML) used to create absorbing boundary conditions. The red dot marks the location of the source in each case. **b**, LDOS spectra for dipoles oriented in-plane, either parallel to the waveguide axis (x), perpendicular to the waveguide axis (y), and out-of-plane (z). The spectra are simulated at the position of the E_y electric field component maximum or the E_x electric field component maximum of the eigenmode of the W1 waveguide, as indicated in **a**. The simulated spectra (gray) exhibit fast, high-amplitude oscillations in the band gap, where emission is strongly suppressed. These artifacts originate from the finite run time of the simulation. To remove the fast oscillations, a second-order Butterworth lowpass with a cutoff frequency of 0.2 times the Nyquist frequency filter is applied to the spectra. The filtered spectra are shown in blue. The dashed horizontal line marks $\rho/\rho_{\mathrm{bulk}} = 1$, separating the regimes of enhancement (above) and suppression (below) of the emission. The vertical red lines indicate the optical transitions into the different crystal field levels of Er:Si in site "A".

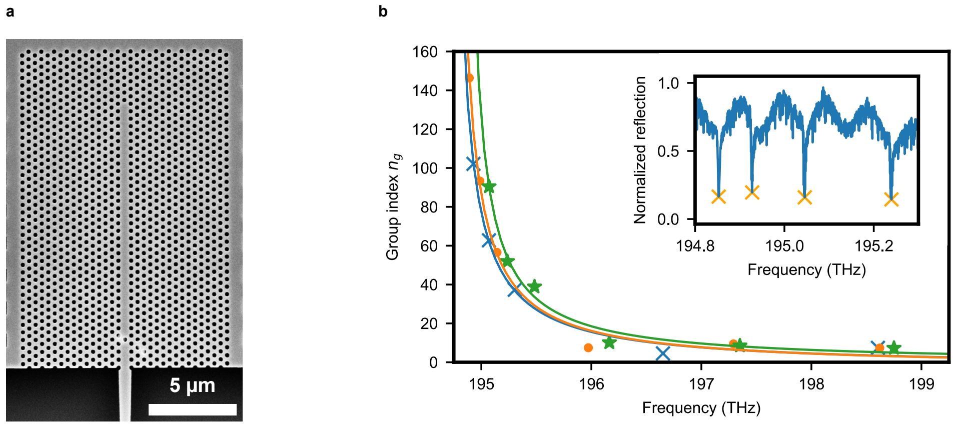

Fig. S2. **a**, Scanning electron microscope image of a PCW in a resonator configuration. A hole is reinserted between fast-light and slow-light section, making this interface partially reflective and thus forming a Fabry-Perot resonator. **b**, The group index is inferred from the free spectral range of the Fabry-Perot resonator. To this end, the reflection spectrum of the structure is measured (inset). The separation between the observed resonances (orange crosses) is then used to determine the group index of waveguide E (main panel). Three different PCWs with identical design show similar group indices but small deviations because of fabrication tolerances: waveguide D (blue crosses), waveguide E (orange circles) and waveguide F (green stars). The data is fit with a reciprocal function (solid lines).

waveguide length. The spectrum for an x dipole at the maximum of E_x looks very similar. Emission is suppressed across the entire spectrum for y dipoles at the E_x maximum and x dipoles at the E_y maximum. The emission of z dipoles is suppressed, independent of the position in the xy plane of the PCW.

II. GROUP INDEX MEASUREMENT

The enhancement of spontaneous emission on the $Y_1 \to Z_1$ transition is directly proportional to the group index of the devices [3], which is thus critical for the modeling and understanding of the devices. Therefore, it is essential to quantify its value and to determine the expected systematic wavelength shift of the devices compared to the simulation. To this end, we fabricate a dedicated additional waveguide next to each structure on the chip. These characterization waveguides feature an extra air hole precisely at the interface of the step coupler and the slow-light section. The effect of the additional hole is to induce a small reflection. Together with the end reflector, a Fabry-Perot resonator is formed whose free spectral range $\Delta\nu$ depends on the group index n_g and the resonator length L:

$$\Delta\nu = \frac{c}{2n_g L}. \tag{1}$$

By extracting $\Delta\nu$ from the reflection spectra, the group index can thus be determined. The results from three PCWs, proximal to the structures used in the main text and with nominally identical hole size and lattice constant, are shown in Fig. S2. The data is fit with a reciprocal function, as the group velocity increases approximately linear in frequency when approaching the band edge of a W1 waveguide [3]. The measurements show that statistical fabrication offsets between the individual devices lead to a slight shift of the bandgap of the PCWs and, as a result, to considerable

variations in the group index at a given frequency. The obtained n_g of up to 140 lead to a significant enhancement of the emission of the wanted $Y_1 \to Z_1$ transition that falls in the slow-light region. For an optimally oriented and positioned two-level emitter in a PCW, this enhancement is

$$F_{\mathrm{P}}^{\max}(\omega) = \left(\frac{3}{4\pi n}\frac{\lambda^2/n^2}{V_{\mathrm{eff}}/a}\right) n_g(\omega), \tag{2}$$

where $n_g(\omega) = c/v_g(\omega)$ is the group index, $V_{\mathrm{eff}} \approx a(\lambda/n)^2/3$ is the effective mode volume, λ the wavelength, n the refractive index, and a the lattice constant [3]. Thus, the decay rate increases linearly with $n_g(\omega)$ and the lifetime of embedded emitters will exhibit a frequency dependence. This effect is shown in Fig. 4 b of the main text. The details of this calculation will be described in the next section.

III. THEORETICAL MODEL FOR THE BRANCHING CONTRAST AND OPTICAL LIFETIME

To predict the effect of the photonic crystal waveguide (PCW) on the branching and lifetime of erbium dopants, a different model is required to predict the effective Purcell enhancement and thus the expected lifetimes, since erbium dopants in silicon have more than one radiative transition. To this end, we assume that in our experiments a $4f$-electron is resonantly excited to the lowest level of the excited state crystal-field (CF) manifold Y_1 and can decay back to one of eight ground state CF levels $Z_1 ... Z_8$ at a rate A_{Z_i}. The bulk lifetime τ is related to the decay constants of the individual transitions via

$$1/\tau = A_{\mathrm{total}} = \sum_{i=1}^{8} A_{Z_i}. \tag{3}$$

In our previous experiments [4], the optical lifetime of the transition in a bulk silicon host was determined to be 142(1) µs, and the fraction of light emitted on the $Y_1 \to Z_1$ transition was $p_{Z_1} =$ 23(5) %. Recording the full emission spectrum in the decay of the Y_1 state by scanning a narrow-band filter now allows determining all individual decay rates:

$$A_{Z_i} = A_{\mathrm{total}} \cdot p_{Z_i}. \tag{4}$$

The resulting lifetime of dopants in the PCW is then given by:

$$\tau'_{\mathrm{total}} = 1/(A_{Z_i} \cdot F_{P,Z_i}), \tag{5}$$

where F_{P,Z_i} are the Purcell factors for each transition. To predict how the lifetime changes with frequency (gray curves in Fig. 4 b), we extract the Purcell factors $F_{P,Y_1\to Z_2}$ to $F_{P,Y_1\to Z_8}$ from the LDOS simulation and make the assumptions that they do not vary significantly between the different spatial positions/dipole orientations and also not for small changes in frequency $<\pm 100$ GHz. The Purcell factor $F_{P,Y_1\to Z_1}$ affecting the lowest-to-lowest transition on the other hand, is computed using Eq. 2 based on the measured n_g of three different waveguides, shown in S2 b. The measured lifetimes and the prediction based on the measured n_g of three identically designed waveguides is shown in Fig. S3.

The minimum lifetime, however, is only achieved for emitters with a matching dipole at the location of maximum LDOS (cf. Fig. S1). However, for Er:Si, the optical dipole moment and its orientation are not known. In addition, the emitters in the studied devices will not be located precisely at the maximum. To account for this, we multiply a factor of 0.217 to the decay on the enhanced transition, F_{P,Z_1}, which is a free factor chosen to give the best agreement of the center gray curve in Fig. 4 b of the main text.

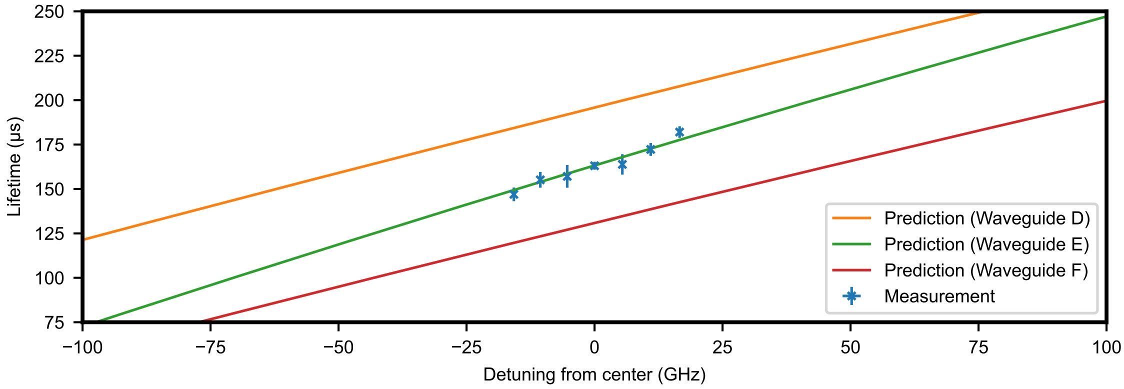

Fig. S3. Frequency dependence of the optical lifetime of the Y_1 level. To calculate the expected lifetime, we measure the group index n_g of three waveguides with identical design and simulate the LDOS inhibition on the $Y_1 \to Z_2$ to $Y_1 \to Z_8$ crystal-field transitions. This model (colored curves) agrees well with the data (blue crosses). In the shown range of detunings, i.e. within ± 100 GHz, the relationship between frequency and lifetime can be approximated by a straight line as $\tau' \propto 1/F_{P,Y_1 \to Z_1}$ and $F_{P,Y_1 \to Z_1} \propto 1/\nu$.

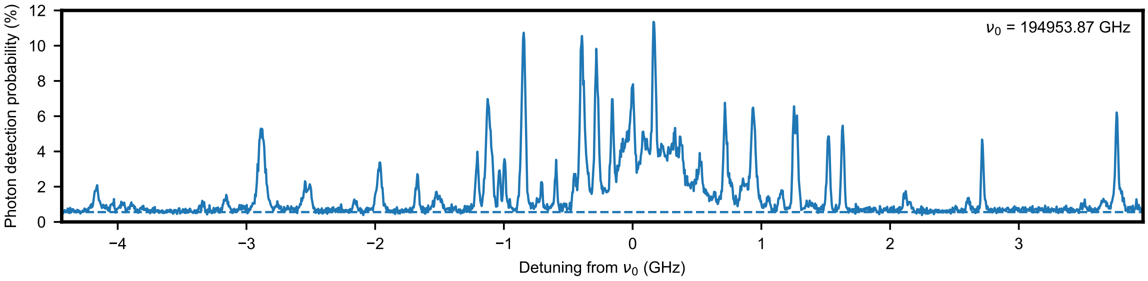

Fig. S4. Fluorescence spectrum measured on waveguide B.

IV. ADDITIONAL FLUORESCENCE SPECTRA

As mentioned in the main text, we investigated three PCWs, A, B and C, with identical parameters except for their lengths. Fig. 3 a in the main text shows the fluorescence spectrum measured on PCW A. For completeness, Fig. S4 shows that of PCW B. Both are qualitatively and quantitatively very similar.

V. PEAK SELECTION AND FITTING

To extract the linewidths in Fig. 3 d of the main text, the relevant peaks in the fluorescence spectra are selected based on their prominence. We fit a Lorentzian emission line profile to all peaks with a prominence of at least 0.2. The resulting fits are shown in Fig. S5 for waveguide A and in Fig. S6 for waveguide B.

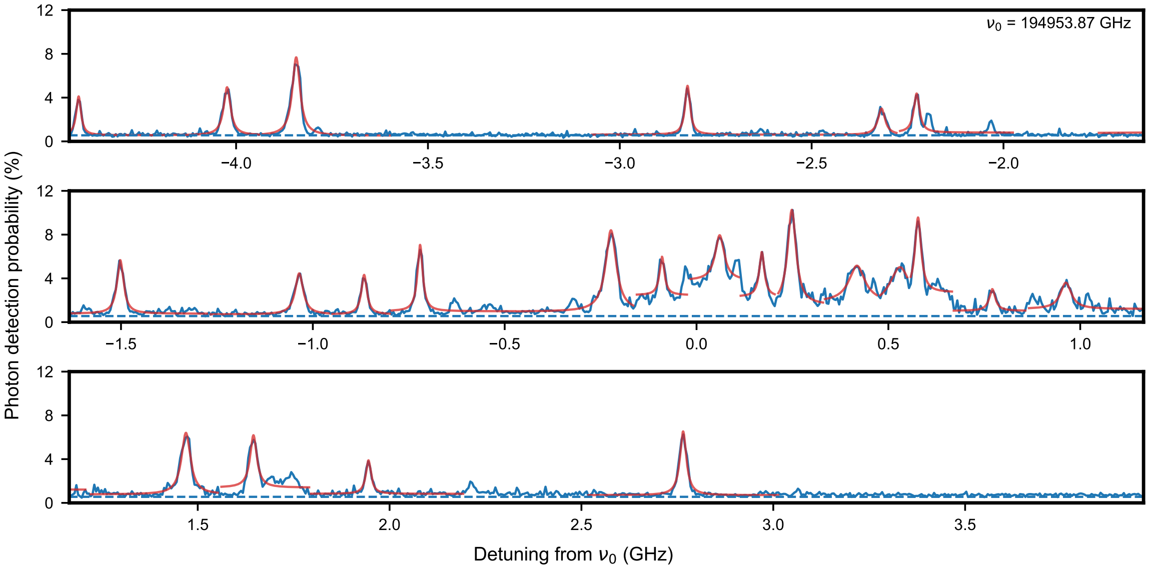

Fig. S5. Fluorescence spectrum measured on waveguide B with automated Lorentzian fits.

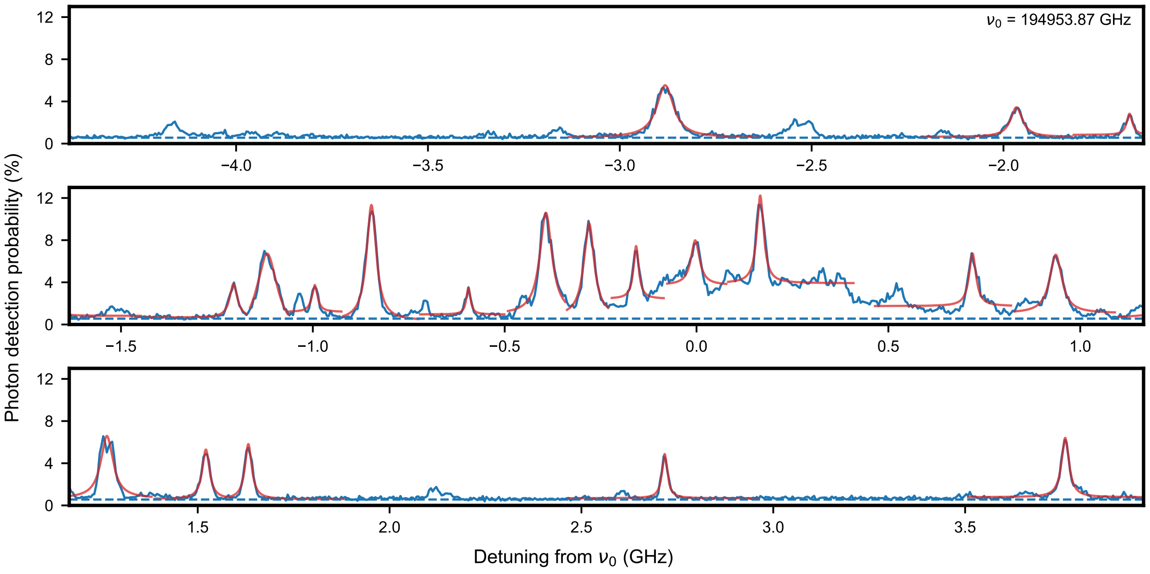

Fig. S6. Fluorescence spectrum measured on waveguide A with automated Lorentzian fits.

VI. FILTER MEASUREMENTS

To determine the branching fraction of the $Y_1 \rightarrow Z_1$ transition, two separate fluorescence spectra are recorded. In both cases, the dopants are excited resonantly to the Y_1 excited-state. Then, the fluorescence photons emitted on the $Y_1 \rightarrow Z_1..Z_8$ CF level transitions are once detected without

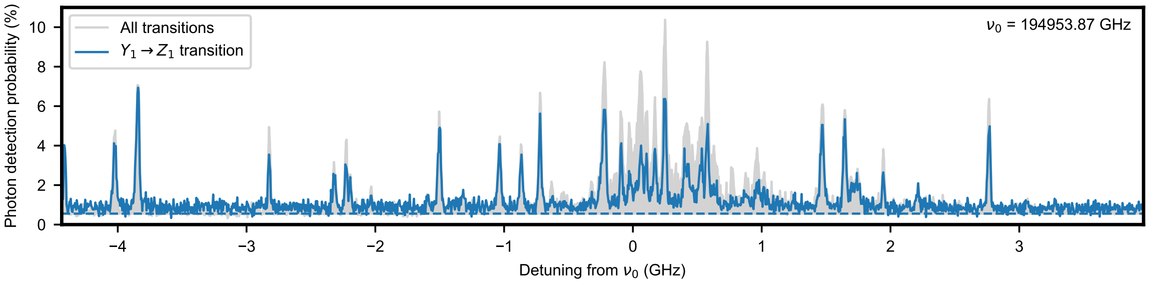

Fig. S7. Fluorescence emission on the $Y1 \to Z1$ transition (blue) and emission on all ($Y_1 \to Z_1 ... z_8$) transitions (gray) after resonant excitation to the Y_1 excited state.

discriminating by frequency and once with a narrow-band filter placed in the detection path that blocks photons emitted on the lower-frequency $Y_1 \to Z_2..Z_8$ transitions. The two spectra are used to determine the branching fraction in Figure 4 c of the main text; they are plotted in Fig. S7 to allow for a comparison.

VII. EXCITATION EFFICIENCY

A rigid upper-bound of the excitation efficiency is required to accurately investigate how the branching ratio between the $Z_1 - Y_1$ transition and the suppressed $Z_i - Y_1$ transitions ($i \in [2, ..., 8]$) is changed in the PCW. This section discusses the details of how this estimate is obtained. In a first step, we determine the characteristic parameters for an emitter coupled to a strong driving field. For this, we measure Rabi oscillations as a function of pulse length on an ion in structure PCW C (see Methods) at frequency 194 954.17 GHz, which provides a good signal-to-noise ratio due to its brightness.

To enable high-power driving, the electro-optic IQ-modulator is removed from the measurement setup for pulsed resonant fluorescence described in Appendix C. We then apply laser pulses of varying duration and measure the subsequently emitted photons to determine the population of the driven ion. The recorded counts integrated over an interval of 600 µs after the pulse are plotted versus different pulse lengths in Fig. S8.

The initial damping of the oscillation, observed for pulse lengths below 0.15 µs, is attributed to amplitude and phase changes caused by the finite bandwidth of the used acousto-optical modulators, which exhibit 6 ns and 25 ns rise times, respectively. Data in this range is excluded from the analysis.

From the data acquired at longer pulse durations, we extract the Rabi frequency by a least-squares fit (solid line in Fig. S8) to a damped Rabi oscillation of the form

$$y(t) = \frac{a}{2}\left(1 - e^{-\gamma t/2}\cos(\Omega t + \phi)\right) + b, \tag{6}$$

where the parameter a rescales to the measurement signal, b accounts for background and detector dark counts, γ is the dephasing, ϕ is a phase shift caused by the finite bandwidth of the pulse shapes, and Ω is the Rabi frequency. The relevant fit parameters are the Rabi frequency $\Omega = 2\pi \times 22.9(2)$ MHz and the dephasing $\gamma = 10(1)$ MHz.

By quantifying the insertion loss of the electro-optic IQ modulator used to obtain the data in Fig. 4 of the main text, and using the square-root scaling of the Rabi frequency with power, we can upper bound the achievable Rabi frequency in this measurement to $\Omega_{\text{max}} \lesssim 86$ MHz. We have compared the Rabi oscillations to those of another dopant on the same structure, which shows a

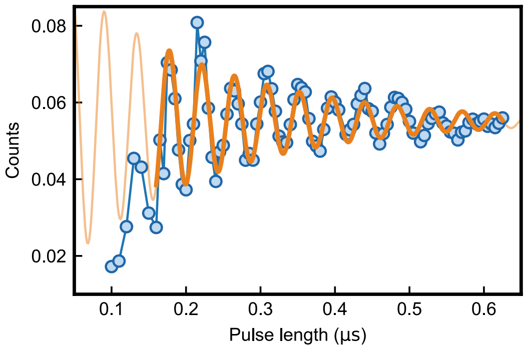

Fig. S8. Pulsed resonant fluorescence with varying pulse length. From the population oscillations observed when driving a single dopant, we extract a Rabi frequency of $\Omega = 2\pi \times 22.9(2)$ MHz and dephasing $\gamma = 10(1)$ MHz using a least-square fit (solid line) of the function defined in eq. 6. Data at pulse lengths below 0.16 µs is excluded from the fit because the pulse amplitude and phase are significantly influenced in this regime by the finite bandwidth of the acousto-optical modulator used for pulsing. An extension of the fit function beyond its range is plotted as a faint solid line.

shorter lifetime (152(7) µs compared to 236(7) µs) but a lower number of collected photons. The extracted Rabi frequency is 30 % lower, indicating the initially considered dopant is well coupled to the driving light field.

The extracted parameters are used to quantify the excitation efficiency of individual dopants in the measurements presented in Sec. IID of the main text. To this end, we model the system using the optical Bloch equations, treating a two-level emitter with density matrix ρ coupled to a strong driving field. Including a damping term γ to reflect the dephasing, we numerically solve the differential equation

$$\frac{d}{dt}\begin{pmatrix}\mathrm{Re}\,[\rho_{12}]\\ \mathrm{Im}\,[\rho_{12}]\\ \rho_{22}-\rho_{11}\end{pmatrix} = \begin{pmatrix}-\gamma & \delta(t) & 0\\ \delta(t) & -\gamma & -\Omega(t)\\ 0 & \Omega(t) & 0\end{pmatrix}\cdot\begin{pmatrix}\mathrm{Re}\,[\rho_{12}]\\ \mathrm{Im}\,[\rho_{12}]\\ \rho_{22}-\rho_{11}\end{pmatrix}. \tag{7}$$

In this calculation, the pulse parameters — its length of 2 µs and the 10 MHz linear chirp of the frequency around the resonance — are included via the temporal dependence of the Rabi frequency $\Omega(t)$ and the detuning $\delta(t)$. The spontaneous emission decay is two orders of magnitude slower than the relevant dynamics and is thus negligible. The initial condition is that the dopant is in the ground state $\boldsymbol{\rho_0} = (0, 0, -1)^{\mathrm{T}}$.

Because of the experimentally observed spectral diffusion, the resonant frequency of the dopants will differ from one repetition of the experiment to the next. To account for this, we solve the differential equation for a range of static offsets δ_0 in the detuning parameter $\delta(t)$. The resulting excited state population is depicted in Fig. S9b. The dopant's spectral diffusion is then incorporated by averaging over the distribution of detunings. It can be estimated by the linewidth measurement $\Delta\nu$ of the ions, which is dominated by dephasing γ and spectral diffusion γ_{sd} such that $\Delta\nu \approx \gamma + \gamma_{sd}$. We simulate according to the measurements discussed in Sec. IIB represented by the mean value $\Delta\nu_{\mathrm{mean}} = 27$ MHz, a one standard deviation lower value $\Delta\nu_{\mathrm{narrow}} = 15$ MHz, and a one standard deviation higher value $\Delta\nu_{\mathrm{broad}} = 39$ MHz. The spectral diffusion follows a Lorentzian distribution

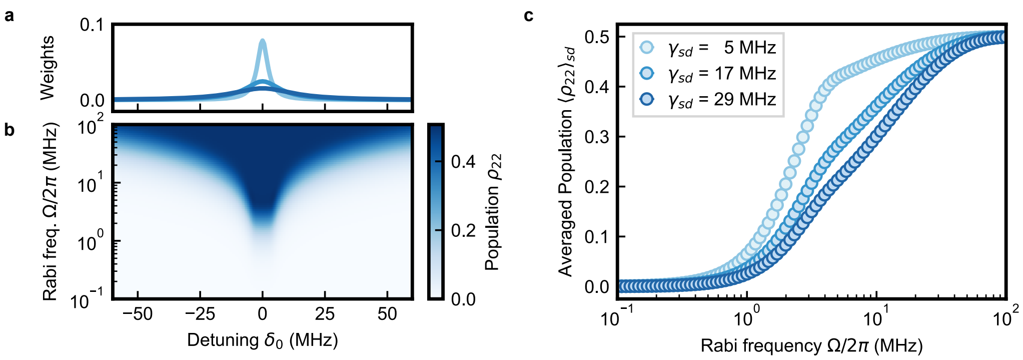

Fig. S9. Simulation of the excitation efficiency upon optical driving. **a**, The dopants exhibit spectral diffusion with a Lorentzian distribution, such that the detuning between laser and dopant needs to be weighted with a corresponding factor when determining the average excitation probability. The shown distributions have the same color and FWHM as in the legend of panel c. **b**, Result of a simulation of the optical Bloch equations, showing the excited state population of a two-level emitter after a pulsed excitation of length 2 µs and a linear chirp of 10 MHz of the driving field frequency around the center frequency. The simulation is performed for various Rabi frequencies Ω and fixed detunings δ_0 from the center frequency. **c**, The averaged population of the excited state of an emitter subject to SD, calculated as a weighted average of values shown in panel a and b, is plotted for three spectral diffusion widths and varying driving field strengths. in the high-power limit, the population approaches, but never exceeds, 50 %.

and we calculate the ensemble average by

$$\langle \rho_{22}(\delta) \rangle_{sd} = \int \frac{1}{\pi\sigma_{sd}\left[1+\left(\frac{\delta}{\sigma_{sd}}\right)^2\right]} \rho_{22}(\delta) d\delta, \tag{8}$$

where $\sigma_{sd} = \gamma_{sd}/2$. We approximate the integral with a weighted sum and appropriate cutoffs as depicted in Fig. S9a. The resulting averaged population in the excited state for various Rabi frequencies is shown in Fig. S9c. For large Rabi frequencies, the population approaches, but never exceeds 50 % for all spectral diffusion parameters. Thus, independent of the precise laser power used, we can give an upper bound for the excitation efficiency of $\eta_{exc} = 50\,\%$.

VIII. SINGLE-PHOTON DETECTION EFFICIENCY

In this section, we rule out that the observed relative increase in $Y_1 \to Z_1$ emission is merely a result of spectral filtering by the PCW. To this end, we calculate an upper bound to the expected number of photons if the $Y_1 \to Z_1$ branching fraction were unchanged compared to those previously determined for erbium dopants in bulk silicon, i.e. $P_{Z1,\mathrm{bulk}} = 23(5)\,\%$ [4].

To this end, we first determine the probability that the emitter is excited after a laser pulse η_{exc}. The used pulses have a length of 2 µs and are thus spectrally more narrow than the spectral diffusion linewidth of ≈ 23 MHz. Still, as we use excitation pulses with a high Rabi frequency, the dopant will also be excited if it is off-resonant with the drive. A numerical simulation based on the optical Bloch equations averaged over the spectral diffusion linewidth leads to $\eta_{exc} \approx 50\,\%$ when assuming fast dephasing, and even lower values for slow dephasing.

Next, to derive an upper bound, we assume that an erbium dopant in the excited state only decays radiatively, and that all emitted photons are coupled into the PCW guided mode with a probability

$\beta \approx 1$ [5].

Finally, we calculate the probability that this photon will eventually be detected after being coupled out of the PCW and transmitted through the optical components of the setup. To this end, we perform an FDTD simulation with MEEP [1] to determine the transmission efficiency of the step coupler and the PCW to strip waveguide interface $\eta_{sc} = 92(2)\,\%$. Furthermore, we measure the maximum reflectivity R of the chips and perform an FDTD simulation to obtain the expected maximum reflectivity of the strip waveguide/step coupler interface $R_{PCW} = 85\,\%$, which allows determining the efficiency of fiber-to-chip (here chip-to-fiber) coupling of $\eta_{ftc} = \sqrt{R} = 73(4)\,\%$. In addition, we calculate the losses in the detection path: The photon is transmitted through an SMF-28 ClearCurve to standard SMF-28 splice and through the cryostat fiber feed-through with a combined probability of $\eta_{ft} = 90(1)\,\%$, through a 95:5 beam splitter with a probability of $\eta_{bs} = 95.0(15)\,\%$, and through an optical switch with a probability of $\eta_{os} = 78(1)\,\%$. If it was emitted on the $Y_1 \rightarrow Z1$ transition, it then passes through a narrow-band filter with a probability of $\eta_{flt} = 47.6(5)\,\%$. Finally, the transmitted photons are detected by the SNSPD with a quantum efficiency of $\eta_{qe} = 75(5)\,\%$.

If the branching fraction in the PCW were unchanged from the bulk value, we would thus expect to detect a photon emitted on the $Y_1 \rightarrow Z_1$ transition with a probability of at most:

$$\begin{aligned} P &= \eta_{exc} \cdot P_{Z1,\text{bulk}} \cdot \beta \cdot \eta_{sc} \cdot \eta_{ftc} \cdot \eta_{ft} \cdot \eta_{bs} \cdot \eta_{os} \cdot \eta_{flt} \cdot \eta_{qe} \\ &= 1.84(43)\,\%. \end{aligned} \tag{9}$$

This is an upper bound as we do not account for propagation losses in the PCW and emission into free space ($\beta < 1$).

The expected experimental value of 3.41(23) % is clearly above the expected value calculated above. This proves that the increase in relative emission on the $Y_1 \rightarrow Z_1$ transition cannot be explained with spectral filtering alone, and instead we observe the effect of the spectrally selective inhibition of radiative decay channels.

---

[1] Oskooi, A. F. *et al.* Meep: A flexible free-software package for electromagnetic simulations by the FDTD method. *Comput. Phys. Commun.* **181**, 687–702 (2010).

[2] Faggiani, R., Yang, J., Hostein, R. & Lalanne, P. Implementing structural slow light on short length scales: the photonic speed bump. *Optica* **4**, 393–399 (2017).

[3] Lodahl, P., Mahmoodian, S. & Stobbe, S. Interfacing single photons and single quantum dots with photonic nanostructures. *Rev. Mod. Phys.* **87**, 347–400 (2015).

[4] Gritsch, A., Weiss, L., Früh, J., Rinner, S. & Reiserer, A. Narrow Optical Transitions in Erbium-Implanted Silicon Waveguides. *Phys. Rev. X* **12**, 041009 (2022).

[5] Javadi, A., Mahmoodian, S., Söllner, I. & Lodahl, P. Numerical modeling of the coupling efficiency of single quantum emitters in photonic-crystal waveguides. *J. Opt. Soc. Am. B* **35**, 514–522 (2018).
